# Prevalence and Antibiotic Resistance of *Staphylococcus aureus* and *Escherichia coli* Isolated from Bovine Raw Milk in Lebanon: A study on Antibiotic Usage, Antibiotic Residues, and Assessment of Human Health Risk Using the One Health Approach

**DOI:** 10.3390/antibiotics11121815

**Published:** 2022-12-14

**Authors:** Maha Hoteit, Joseph Yaghi, Andre El Khoury, Rouaa Daou, Pamela Hindieh, Jean Claude Assaf, Jana Al Dawi, Jennifer El Khoury, Ayoub Al Jawaldeh

**Affiliations:** 1 Faculty of Public Health, Lebanese University, Beirut P.O. Box 6573, Lebanon; 2 PHENOL Research Group (Public HEalth Nutrition Program Lebanon), Faculty of Public Health, Lebanese University, Beirut P.O. Box 6573, Lebanon; 3 Lebanese University Nutrition Surveillance Center (LUNSC), Lebanese Food Drugs and Chemical Administrations, Lebanese University, Beirut P.O. Box 6573, Lebanon; 4 Lebanese University Medical Center, Lebanese University, Beirut P.O. Box 6573, Lebanon; 5 Laboratoire de Mycologie et Sécurité des Aliments (LMSA), Faculty des Sciences, University Saint-Joseph de Beyrouth, Campus des Sciences et Technologies, Mar Roukos, Matn, Beirut P.O. Box 6573, Lebanon; 6 World Health Organization Regional Office for the Eastern Mediterranean, Cairo 11371, Egypt

**Keywords:** raw milk, *Escherichia coli*, *Staphylococcus aureus*, antibiotic-resistant bacteria, antibiotic residues, risk characterization, Lebanon

## Abstract

The emergence, persistence, and spread of antibiotic-resistant microbes is a tremendous public health threat that is considered nowadays a critical One Health issue. In Lebanon, the consumption of raw bovine milk has been recently reported as a result of the financial crisis. The objectives of the current study were (1) to evaluate raw bovine milk samples in a comprehensive manner for the types of antibiotics used and their residues, (2) to determine the presence of mesophilic bacteria, extended-spectrum β-lactamase (ESBL)-producing *Escherichia coli* and methicillin-resistant *Staphylococcus aureus* (MRSA), and (3) to determine the associated human health risk caused by drinking raw milk with antibiotic residues among all age categories. LC-MS-MS was used to carry out the analysis. From 200 milk samples, 30 (15%) were found contaminated with four major antibiotics. The highest average concentration detected was for oxytetracyline 31.51 ± 13.23 μg/kg, followed by 5.5 ± 0.55 μg/kg for gentamicin, 4.56 ± 0.73 μg/kg for colistin, and 4.44 ± 0.89 μg/kg for tylosin. The mean contamination among most samples was below the maximum residue limits (MRLs). Upon comparison with the acceptable daily intake (ADI), the estimated daily intake (EDI) across all age groups was acceptable. The hazard quotient (HQ) was also below 1 across all age groups, signifying the absence of associated health risks for the Lebanese consumers. On the other hand, all milk samples were found exceeding the maximum tolerable value of mesophilic flora. Antibiotic-resistant bacteria (ARB) were detected and represented by ESBL-producing *E. coli* and MRSA isolates. Thus, the greatest threat of antibiotic use in Lebanon does not fall under antibiotic residues but rather the proliferation of antibiotic resistance in potentially pathogenic bacteria. In this study, the virulence profile of detected bacteria was not investigated; thus their pathogenicity remains unknown. Therefore, to mitigate this health threat in Lebanon, a “One Health” action plan against ABR is required. It will provide a framework for continued, more extensive action to reduce the emergence and spread of ABR in Lebanon.

## 1. Introduction

Antibiotic resistance (ABR) is a pandemic that is considered as one of the top 10 threats intimidating humanity with the potential to lead to 10 million deaths per year by 2050 [1]. ABR in humans is greatly associated with ABR in farm animals [2]. Not only does it include the transmission of antibiotic-resistant bacteria (ARB) to humans through dairy products or milk but also residues of the drugs themselves [2].

In Lebanon, dairy farming is considered to be the second-highest share of all agri-food establishments, with about 100 dairy factories distributed mainly in the Beqaa (45%) and Mount Lebanon regions (31%) [3]. Furthermore, milk production and the food supply quantity of milk and whole fresh cow milk have increased considerably over the past few years in Lebanon [4,5]. The average milk consumption among the Lebanese population was reported to fluctuate between 26.65 g/day and 813.4 g/day [6,7,8,9].

Over the past twenty years, an enormous number of articles and reviews addressing the microbiological quality, ARB profile, along with antibiotic residues in raw cow’s milk have been published [10]. In reference to the literature, bacteriological standards for raw bovine milk could be found [11]. However, due to various reasons including the particularities of milk handling and production, as well the enormous contamination risks, it is often difficult to consistently meet these standards [12]. Moreover, upon worldwide evaluation of dairy, specifically raw milk, *Staphylococcus aureus (S. aureus)* and *Escherichia coli (E. coli)* were found to be the principal pathogens contaminating milk [13]. In addition, in the agro-food sector, mastitis, one of the leading causes of economic losses in the dairy industry [14], has become more prominent, which has promoted the increased use of antibiotics in the dairy sector [15]. 

Beyond mastitis, antibiotics are also used in food producing animals for the prevention and treatment of various infectious diseases common in dairy cows, such as respiratory, urinary, and foot infections, and in other cases as feed proficiency enhancers and growth promoters, as many reports have linked the latter uses with economic gains [10]. In some countries including Lebanon [16], where strict regulations are absent, antibiotics are still being used as growth promoters [17]. 

Antibiotic sales and utilization among farm animals are expected to increase globally [18]. This is especially critical, as over the years, both *S. aureus* and *E. coli* have developed resistance to many antibiotics, leading to the phenomenon of multidrug resistance (MDR) [19].

According to a recent systematic review, *S. aureus* was found to have a high resistance against penicillin and many other antibiotics [20]. Not only that, but also the overuse of β-lactam antibiotics along with the emergence of β-lactamases during the treatment of infections due to *S. aureus* reveals that one of the viable risks to human health includes the possibility of horizontal transfer of resistance genes [21]. This raises concern as methicillin-resistant *S. aureus* (MRSA) isolates have been increasing over the years, and their prevalence has been found to be the highest in Asia [22]. Furtherly, it is well known that β-lactamases, including extended-spectrum β-lactamases (ESBLs), are easily transferable among different bacteria, and previous studies reported the detection of ESBL-producing *E. coli* isolates in raw milk [23,24]. 

Until now, close inspection of such issues has been relatively mediocre, both nationally and globally. Although improving, accomplishing better antibiotic stewardship on the farm is challenging as “antibiotics are an integral part of industrial agriculture and there are very few alternatives” [10]. Thus, the potential transmission of such resistant microorganisms, along with the drugs themselves to humans through the food chain, has become a growing concern among health authorities [25,26,27,28,29]. 

Nowadays, a unified global effort led by the World Health Organization (WHO) in collaboration with the Food and Agriculture Organization of the United Nations (FAO), the World Organization for Animal Health (OIE), and United Nations Environment Programme (UNEP), yielded a strategic framework for collaboration on antibiotic resistance to advance a “One Health” response to ABR at the global, regional, and country levels [30]. 

In Lebanon, food safety issues including antibiotic resistance are considered a priority especially due to the current political instability and financial crisis. The consumption of raw bovine milk has been recently reported among all age categories. This can be primarily attributed to the shortage of infant formula [31] and the increase in the price of processed milk [32]. Therefore, for the purpose of consumer health surveillance, we conducted this study (1) to evaluate comprehensively the types of antibiotics available in raw cow’s milk and their residues; (2) to determine the presence of mesophilic bacteria, extended-spectrum β-lactamase (ESBL)-producing *E. coli*, and methicillin-resistant *S. aureus* (MRSA); and (3) to determine the associated human health risks for all age categories in Lebanon using a One Health approach.

## 2. Materials and Methods

### 2.1. Ethics Statement

This project was carried out with the cooperation of the Lebanese Ministry of Agriculture (MOA) and Saint Joseph University in Lebanon in 2019/2020. Approval was granted from the MOA in Lebanon for the collection of raw milk samples from the representative farms, and the collection of samples was performed in accordance with the national norms for animal sampling and manipulation. The selection of farms was based on their geographical location, the presence or absence of a nearby community, and their overall size (number of cows per farm). 

### 2.2. Questionnaire-Based Interviews with the Farm Stakeholders

Before the samples were collected, the head of the allocated farms were subjected to various questions interrogating the health of the cattle and the diseases they might be suffering from. All answers were recorded and taken into consideration for the purpose of assessing the health of the cattle.

### 2.3. Sample Collection 

**Figure 1:**
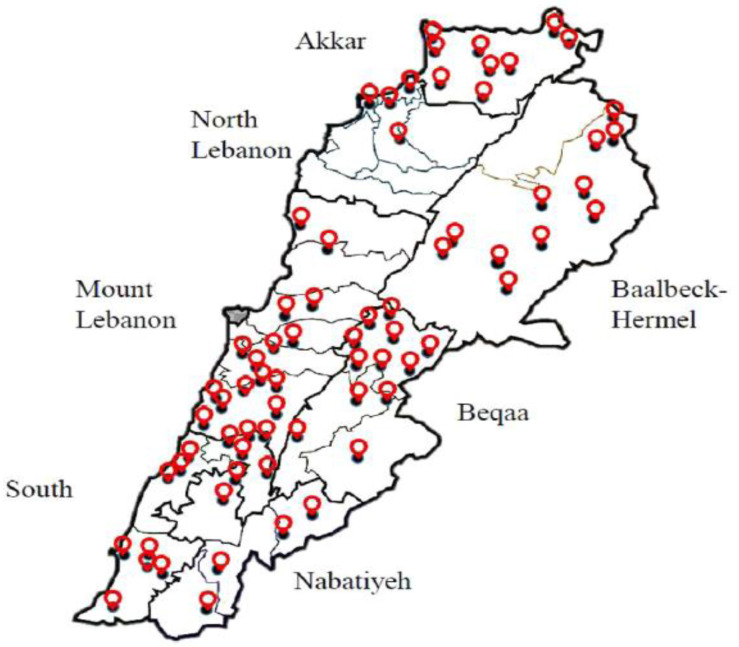
Sampling points of raw milk across Lebanon.

Sampling was conducted between April 2018 and September 2019 at regular time intervals, bimonthly, during the whole year. An average of five to six samples were collected from each farm. Throughout the project, 15 inspectors from the MOA were given theoretical and applied training on collecting raw milk samples. Following the training, a total of 400 pooled samples of raw milk were collected from the milk storage tanks after milking the herds from 70 small and large farms in all Lebanese governorates, except Beirut (Figure 1). The governorates Akkar and Nabatieh were added to the North Lebanon and South Lebanon regions, respectively (Table 1). Technical assistance, i.e., gloves, costumes, and a portable refrigerator, were provided by the MOA team. All milk samples were collected into sterile sampling bottles. About 1000 mL of milk were taken from each farm. All milk samples were placed in falcon tubes with proper labelling for each sample and transported in a cooler sampling case to the microbiology laboratory at the faculty of Sciences of Saint-Joseph University of Beirut. Finally, all samples were stored in a deep freezer at −20 °C. The duration of time between the collection of samples and deep freezing ranged between 30 and 45 min. Due to the limited budget, out of the 400 samples collected, only 200 samples were tested. Farms representativeness was respected during sample selection. 

### 2.4. Determination of Antibiotic Residues 

#### 2.4.1. Dairy Sample Preparation and Analysis of Antibiotics Using the Liquid Chromatography with Tandem Mass Spectrometry (LC-MS-MS) Method 

Milk samples were shaken vigorously. Each sample (1 g) was then weighed into a 5 mL polypropylene tube. Acetonitrile (1 mL) was added to the sample, which was then shaken for 5 min on the vortex and then centrifuged at 12,000 rpm for 5 min for the removal of protein. The supernatant was filtered through a nylon micro filter (0.45 pm pore size) directly into the LC vial, and sixteen residue assays for the commonly used veterinary antibiotics in Lebanon—enrofloxacin, marbofloxacin, trimethoprim, gentamicin, sulfamethazin, oxytetracycline, florfenicol, tilmicosin, spectinomycin, penicillin G, amoxicillin, spiramycin, colistin, kanamycin, danofloxacin, and tylosin—were carried out using liquid chromatography with tandem mass spectrometry (LC-MS-MS).

Stock solutions were prepared by dissolving 10 mg of the standard in 10 mL of methanol. This solution was further diluted using the same solvent to make a standard working solution of different concentrations. This solution was stored at a temperature of 20 °C. 

#### 2.4.2. Liquid Chromatography Conditions

The analysis was performed using a Thermo Scientific TSQ Fortis triple quadrupole mass spectrometer.

Analytical column: Thermo Scientific^TM^ Hypersil Gold (100 × 2.1 mm, 1.9 pm).

Mobile phases:

A = 1 mM heptafluorobutyric acid and 0.5% formic acid in water
B = 0.5% formic acid in acetonitrile/methanol (1/1)
C = 2% methanol in water
D = acetone/acetonitrile/isopropanol (20/40/40).

Cleaning solvents for the auto-sampler:

Solvent 1: acetonitrile/water (20/80)
Solvent 2: acetone/acetonitrile/isopropanol—20/40/40

#### 2.4.3. Mass Spectrometry Conditions

Spectrometric analysis was carried out using a TSQ Quantum Access MaxTM triple quadrupole system. Data acquisition for quantification and confirmation was performed in MRM mode. All selected reaction monitoring (SRM) traces (parent, qualifier, and quantifier ions) were individually tuned for each target analyte by the direct injection of the individual working standard solution (10 mg/mL). Data acquisition and processing was performed using Thermo Scientific Xcalibur software. Ionization mode: electrospray (ESI); scan type: SRM; polarity: positive ion mode; spray voltage (V): 3500; ion sweep gas pressure (arb): 0; vaporizer temperature (°C): 400; sheath gas pressure (arb): 50; aux gas pressure (arb): 10; capillary temperature (°C): 370; collision gas pressure (mTorr): 0; cycle time (s): 0.6 peak width: QI/Q3 the full width of a peak at half its maximum height (FWHM) of 0.70 Da. The parameters for SRM analysis (precursor ion, quantifier ions, collision energy for quantifier ion, and tube lens) for targeted compounds were collected from the literature and LC-MSMS Thermo Scientific Applications for Food Safety Analysis. The CCα (µg.kg^−1^) decision limit, CCβ (µg.kg^−1^) detection capability, recovery %, CV intraday %, and CV interday % for antibiotics are presented in Table 2.

### 2.5. Estimation of Hazard Quotient and Exposure Risk Assessment among All Age Categories

#### 2.5.1. Risk Assessment

The probability of potential adverse health effects caused by antibiotic residues in raw milk was measured by calculating the risk assessment.

##### Acceptable Daily Intake (ADI) and Maximum Residue Limits (MRLs) of Antibiotics

The acceptable daily intake (ADI) is determined as a conservative estimate of the safety ingestion levels by the human population based on the lowest “no effect level” (NOEL) among a battery of toxicological safety studies. The ADI values set by the Codex Alimentarius Commission (CAC) vary between a concentration of 0–30 μg/kgBW/day for oxytetracycline and tylosin, 0–7 μg/kgBW/day for colistin, and 0–20 μg/kgBW/day for gentamycin based on a microbiological end-point derived from in vitro minimum inhibitory concentration (MIC) susceptibility testing and fecal binding data (MICcalc = 1.698) [33]. In raw bovine milk, the MRLs of oxytetracycline, colistin, gentamycin, and tylosin, expressed in ug/kg, were equal to 100 [34], 50, 200, and 100, respectively [33].

##### Exposure Assessment of Antibiotic Residues in Milk

Exposure assessment was conducted using both the deterministic (point estimate) and probabilistic distribution-based or population-related approaches across four target age groups, which included early childhood, middle childhood and adolescents, adults, and elderly. Per capita milk consumption was obtained from FAOSTAT and was reported as 110.95 g/per capita/day and was used in the deterministic exposure assessment [5]. The results obtained from recently performed Lebanese nutrition surveys were used as a probabilistic exposure estimation to derive the average milk intake among the Lebanese population studied [6,7,8,9]. 

To calculate the estimated daily intake (EDI) that shows the levels of exposure to the antibiotic residues, the average daily milk intakes among all age categories, as reported by multiple national representative surveys [6,7,8,9], were multiplied by the mean residue concentration obtained in this study and divided by the average weight of each population being studied [35]. Those values were then taken into consideration to declare the level of exposure of the Lebanese population in both genders and across all ages.

Equation (1):

EDI (µg/kg BW/day) = Σ[Daily dairy intake (kg/person/day) × Mean residue concentration (µg/kg)] ÷ BW (kg).(1)

The values obtained from this equation, which reveal the level of exposure of the Lebanese population to the antibiotic drug residues in the raw milk, were then compared to ADI [33]. To further investigate the risk associated with the antibiotic residues in milk, the following equations were used [35]: 

Equation (2):

Intake of antibiotic residue (µg/day) = Mean residue concentration (µg/kg) × daily milk consumption (kg/day)(2)

Equation (3):

%ADI = 100 × Intake (µg/day)/[ADI (µg/kgbw/day) × Body Weight (kg)](3)

#### 2.5.2. Assessment of Hazard Quotient (HQ)

To characterize the health risks associated with the dietary exposure to antibiotics through milk, the hazard quotient (HQ) was calculated. The HQ is the ratio of the potential exposure concentration to a substance and the level at which no adverse effects are expected [36]. Depending on the mean concentration of antibiotic residues detected in milk samples, the risk was assessed on the basis of the concentration of ADI recommended by JECFA [37]. If the observed HQ ratio was below or equivalent to 1, the potential effects on the human health upon exposure to the substance were not considered, as no adverse effects are likely to occur. On the other hand, if HQ was higher than 1, it was considered as a risk to the consumers and a possible cause of harm to the human health [36]. 

Equation (4) [36]:

Hazard Quotient = Estimated Daily Intake (EDI)/Acceptable Daily Intake (ADI)(4)

### 2.6. Isolation of Antibiotic-Resistant Bacteria

#### 2.6.1. Sample Preparation

Serial ten-fold dilutions of raw milk samples were prepared using tubes containing 9 mL of sterile peptone water (up to 1:10,000 dilutions). Then, 1 mL of each sample dilution were cultured on plate count agar (PCA). 

#### 2.6.2. Evaluation of the Number of the Mesophilic Flora according to NF ISO 4833

From each dilution, 1 mL was transferred to a Petri dish, and 15 mL of the TSA agar at 47 °C was poured into each of the dishes. The inoculum was then thoroughly mixed with the medium. After the solidification of the media, the Petri dishes were turned over. Finally, the dishes were incubated for 72 h ± 3 h at 30 °C.

#### 2.6.3. Evaluation of the Number of *E. coli* according to the RAPID’ *E. coli* Method NF SDP07/1-07/92

From each dilution, 1 mL was transferred to a Petri dish, and 15 mL of RAPID’ *E. coli* agar was poured into each of the dishes. The inoculum was then thoroughly mixed with the medium. After solidification, the Petri dish was turned over and incubated for 24 h at 42 ℃. After incubation, all violet to pink colonies were counted.

#### 2.6.4. Evaluation of the Number of *S. aureus* Coagulase Positive ISO6881-1

*S. aureus* was detected in the samples using Baird–Parker agar with sterile egg yolk. After incubation of plates at 37 °C for 24 h, typical black colonies with a clear and opaque zone due to proteinase and lecithinase and the tellurite reduction were considered as presumptive *S. aureus*. 

#### 2.6.5. Confirmation of *E. coli* and *S. aureus*

The *E. coli* isolates were confirmed by the indole urea test. Similarly, *S. aureus* isolates were confirmed by biochemical tests such as coagulase, catalase, DNase, lecithinase, oxidase, lysostaphin sensitivity, VP, urease, glucose, and mannitol fermentation.

#### 2.6.6. Antibiotic Resistance of *E. coli* and *S. aureus* Isolates Using the Disk Diffusion Method

All *E. coli* and *S. aureus* isolates were tested for antibiotic resistance. Testing was carried out by using the disk diffusion method (Kirby–Bauer method) according to the guidelines of the Clinical and Laboratory Standards Institute (CLSI) (Performance Standards for Antibiotics Susceptibility Testing, 2017). The susceptibility testing was done using nine different commonly used antibiotics for the treatment of human infections. These antibiotics purchased from Sigma Aldrich (Darmstadt, Germany) were the following: Fep: cefepime 30 µg, TC: ticarcillin 75 µg, CTX: cefotaxime 30 µg, AMC: augmentin 20 µg, CAZ: ceftazidime 30 µg, TZP: piperacillin-tazobactam 36 µg, P: penicillin 10 IU, FOX: cefoxitin 30 µg, ETP: ertapenem 10 µg, IMI: imipenem 10 µg. These disks were placed on the surface of an agar plate previously inoculated with the bacterial strain, and the plates were incubated at 37 °C for 18 h. After the incubation period, zones of inhibition were recorded using a ruler to the nearest millimeter (mm) and classified as resistant (R), intermediate (I), or susceptible (S) based on the guidelines of the Clinical and Laboratory Standards Institute (CLSI) (Performance Standards for Antibiotics Susceptibility Testing, 2017).

## 3. Results

### 3.1. Questionnaire-Based Interviews with the Farm Stakeholders

On the farms, the cattle were found to suffer from various diseases, which also varied among the governorates. For example, cowpox–septicemia and hoof disease were highly prevalent in Mount Lebanon, more specifically in Jbeil (Mount Lebanon), while hepatitis, mastitis, and hepatitis–mastitis–respiratory infections were more prevalent in Tyre (South Lebanon). On the other hand, FMD (foot and mouth disease) and mastitis–hoof disease were more common among the cattle in Beqaa and Baalbeck–Hermel governorate. Further details are shown in Table 3.

### 3.2. Antibiotic Residues

In this study, among the 200 samples screened, 30 (15%) showed residual levels of four major antibiotics, namely, oxytetracycline, colistin, tylosin, and gentamicin. The concentrations of the mentioned antibiotic residues, presented as mean and ranges, are shown in Table 4. The obtained results showed that oxytetracycline was one of the most used antibiotics, as it had the highest concentration compared to all other antibiotics, with a mean residue level of 31.51 μg/kg. In addition, the mean residual level of oxytetracycline was approximately 5.7 times that of gentamicin, which had a mean concentration of 5.5 μg/kg. On the other hand, colistin and tylosin had similar concentrations, with means of 4.56 μg/kg and 4.4 μg/kg, respectively. Overall, the mean contamination in the 30 positive samples was below the MRLs set by the FAO/WHO and EU, except for one sample representing 3.3% of all samples, which recorded a mean residue level exceeding the MRL for oxytetracycline (100 μg/kg), as shown in Table 4.

The prevalence of samples contaminated with antibiotic residues varied greatly among the various governorates in Lebanon. The Beqaa and Baalbeck–Hermel governorate showed the highest prevalence of antibiotic residues, with 22 out of the 30 contaminated samples (73.3%) belonging to this governorate alone. On the other hand, in Mount Lebanon, four samples (13.25%) were contaminated with antibiotic residues, which is double that found contaminated in North and South Lebanon, with each having two contaminated samples (6.7%). Further details are demonstrated in Table 5.

### 3.3. Risk and Exposure Assessment

In this study, exposure assessment was performed using both deterministic and probabilistic approaches for the four age groups targeted, which included early childhood, middle childhood and adolescents, adults, and elderly.

#### 3.3.1. Deterministic Approach

Oxytetracycline, which was found to be the most prevalent antibiotic residue, recorded the highest EDI average among all age groups compared to the other detected antibiotics. Among the early childhood age group, the values ranged between 2.607 and 2.742 μg/kg BW/day for males and females, respectively. Those values were the highest compared to all other age groups. For example, the mean EDI of oxytetracycline in the middle childhood and adolescents age group was 0.672 and 0.723 μg/kg BW/day for males and females, respectively, which is slightly higher than that of the adults and elderly, which reported values of 0.583 μg/kg BW/day for both adult males and females, and 0.443 and 0.488 μg/kg BW/day for elderly males and females, respectively. Regarding colistin and tylosin, the average EDI for both genders was significantly similar among all age groups, as shown in Table 6. On the other hand, those values were slightly lower than those of gentamicin, the average EDI values of which were 0.455 and 0.479 μg/kg BW/day for males and females, respectively, in the early childhood age group. These values were approximately four times higher than those reported by the middle childhood and adolescents age group, with values of 0.117 and 0.126 μg/kg BW/day for males and females, respectively. Regarding the adults age group, the mean EDI of gentamicin recorded a value of 0.102 μg/kg BW/day for both males and females, which was higher than those of the elderly, that had average EDI values of 0.077 and 0.085 μg/kg BW/day for males and females, respectively.

#### 3.3.2. Probabilistic Approach

The mean EDI of oxytetracycline ranged between 1.619 and 1.703 μg/kg BW/day for males and females in early childhood. This value was higher than that of the middle childhood and adolescents age group, which reported an average EDI of 0.037 and 0.040 μg/kg BW/day for males and females, respectively. Both adults and elderly had significantly similar EDI values, with averages ranging between 0.014 and 0.019 μg/kg BW/day for adult males and females, and 0.013 and 0.020 μg/kg BW/day for elderly males and females, respectively.

All details are presented in Table 7. Regarding colistin, tylosin, and gentamicin, the average EDI in both genders across different age groups reported was significantly similar. For example, among the early childhood age group, the EDI mean of the three antibiotics ranged between 0.228 and 0.283 μg/kg BW/day for males and 0.240 and 0.297 μg/kg BW/day for females. Furthermore, both colistin and tylosin had the same average EDI of 0.005 and 0.006 μg/kg BW/day for middle childhood and adolescent males and females, which was slightly lower than that of gentamicin, which reported values of 0.006 and 0.007 μg/kg BW/day for males and females, respectively. As for the remaining age groups (adults and elderly), all three antibiotics recorded the same EDI averages of 0.002 and 0.003 μg/kg BW/day for males and females, respectively.

Upon comparing the average EDI among all age groups using both approaches, it was found that the early childhood age group had the highest risk of exposure, not only to oxytetracycline, but also to the other three groups of antibiotics detected in milk. Fortunately, the EDIs reported in this study were all found to be lower than the set ADI, as demonstrated in Table 6 and Table 7. Furthermore, in this study, all EDIs obtained through the deterministic approach were found to be exceeding those estimated using the probabilistic approach.

As per the mean concentration of the antibiotic residues obtained, the HQ was calculated to characterize the risk of dietary exposure to oxytetracycline, colistin, tylosin, and gentamicin through the consumption of milk, and to estimate any health threats to the consumers. The maximum level of the acceptable daily intake (ADI) of the four antibiotics established by JECFA [33] was taken into consideration upon HQ calculation.

In the present study, in addition to the EDI of all residues being below the ADI, upon calculation using the deterministic and probabilistic approach, the HQ among all age groups for the four major groups of antibiotic residues detected was below 1 (Table 6 and Table 7).

### 3.4. Isolation of Antibiotic-Resistant Bacteria

Upon bacterial analysis, the milk samples showed a high prevalence of mesophilic flora where all samples (100%) showed contamination above the permitted maximum value of raw milk according to the Lebanese norm (LIBNOR) ranging from 10^4^ to 10^5^ CFU/mL [45]. Table 8 shows the prevalence of microbial contamination by mesophilic flora, *E. coli*, and *S. aureus* of raw milk samples with antibiotic residues according to the farm locations, and the number and the health issues of cattle per farm.

As for *Escherichia coli*, four samples (13.3%) were found to be contaminated and exceeded the maximum tolerable value set by LIBNOR (10^2^ to 10^3^ CFU/mL), with the concentration ranging between 2 × 10^2^ and 4 × 10^2^ CFU/mL. These isolates were detected in four “specialized dairy cattle farms” (number of cattle more than 7) located in Beqaa/Hermel governorate (one from West Beqaa, one from Hermel, and two from Baalbeck).

Concerning *S. aureus* isolates, three samples (10%) were found to be exceeding the maximum tolerable value set by LIBNOR (10 to 10^2^ CFU/mL) [45]. These samples were derived from three “specialized dairy cattle farms” (two in Beqaa and one in Mount Lebanon). However, the concentration of *S. aureus* in the contaminated samples was remarkably higher than that of *E. coli*, as it reported values ranging between 160 and 8.4 × 10^2^ CFU/mL. According to Table 8, it appears that the cattle in all the governorates were treated with antibiotics without being sick or showing any disease symptoms. Furthermore, higher antibiotic use and residues was observed on farms with high numbers of cattle and located in the Beqaa–Hermel governorate (*p* = 0.007). Furthermore, it was found that a farm with 70 cows presented with two MDR antibiotic-resistant bacteria (Table 8).

### 3.5. Antibiotic Susceptibility Testing

After isolating *E. coli* from contaminated milk samples, the antibiogram showed the presence of a characteristic “champagne cork” image of synergy between the discs of the four different antibiotics, which included CTX (cefotaxime), CAZ (ceftazidime), FEP (cefepime), and AMC (augmentin), indicating the occurrence of an ESBL-resistant strain of *E. coli* in this milk sample. Therefore, this isolate was considered to be a multidrug-resistant (MDR) strain. Other milk samples, were found only resistant to one of the antibiotics; thus, they were not considered as MDR. For example, isolates B143 and B27 were resistant to Tic (ticarcillin), while sample B51 was resistant to both Tic (ticarcillin) and AMC (augmentin).

As for *S. aureus*, the antibiogram showed that the isolate from sample B234 was resistant to both P (penicillin) and FOX (cefoxitin) with 1 mm inhibition zone for penicillin (<10 mm) and 18 mm for cefoxitin (<22 mm). This revealed the occurrence of a methicillin-resistant *S. aureus* (MRSA) strain. On the contrary, the two other *S. aureus* isolates were sensitive to the same antibiotics.

## 4. Discussion

In the current study, among the 200 samples screened, 15% showed residual levels of four major antibiotic groups, including oxytetracycline, colistin, tylosin, and gentamicin. Overall, the mean contamination among the positive samples was below the MRLs set by the FAO/WHO and EU, except for one sample that recorded a mean residue level exceeding the MRL for oxytetracycline. In comparison to the ADI, both the EDI and HQ values showed no health risk for Lebanese consumers. However, the availability of ARB represented by ESBL-resistant *E. coli* and MRSA isolates presented a challenge for the same consumers.

Our findings show that the cattle in many governorates were treated with antibiotics without being infected. Moreover, higher antibiotic use and residues were observed on farms with high numbers of cattle and located in the Beqaa–Hermel governorate. Consequently, the high number of cattle per farm has led to a higher appearance of antibiotic residues and in return the emergence of antibiotic-resistant bacteria. This is not surprising, as the Beqaa valley is considered the center of Lebanon’s dairy industry, with around 75% of cows in Lebanon being raised in this region alone [3].

Our findings are supported by Dankar et al., who showed that among the Beqaa valley farmers, high practices of self-administration of antibiotics were observed, in addition to those who prescribe antibiotics by themselves, which is critical and can lead to underdosing or overdosing in dairy cattle without perceiving whether the antibiotics course was completed or not. Non-compliance by dairy farmers could also be explained by the fear of financial loss that may arise from discarding milk during the withdrawal period. In addition, in the same study by Dankar et al., the majority of farmers stated that antibiotics misuse occurs only when delivered as an overdose to sick cows, reflecting the attitude of preferring to provide an underdose of an antibiotic, believing this to be one of their good practices. Significantly, underdosing was reported by many scientists as being the main reason for dangerously developing ABR. In addition, pathogens exposed to subinhibitory concentrations of the antibiotics will lead to more survival of bacterial populations in that animal, generating an advanced resistance to the antibiotics [47].

Although frequently recognized as an important source of food-borne disease outbreaks, raw milk is still being consumed in low-income countries, including Lebanon, which has been facing an economic crisis in the last two years. In addition, after the explosion of Beirut’s port in 2020 and the Russia–Ukraine war, the access to infant formulas for the importation-dependent Lebanon has become very limited [48]. Other consumer products such as milk powder were also missing from the supermarket shelves [49]. As a result, Lebanese people living in rural and urban areas developed a new habit of drinking raw bovine milk since it is cheaper, and they believe that it is more beneficial for their health. However, many reliable sources agree that the pasteurization of milk is mandatory to reduce associated health risks [50,51,52,53,54,55]. In addition, research has shown that there is no nutritional difference between pasteurized and raw milk [56]. Some people tend to boil raw milk before consuming it. However, this is not an optimal solution, as not all of them follow the correct milk boiling practices. In addition, boiling milk at a high temperature for a prolonged period of time can cause considerable deterioration in its nutritional content [56].

On the other hand, the continuous and cumulative consumption of milk with antibiotic residues can have adverse effects on the human health. Some of these toxic effects include allergy, immunopathological effects, carcinogenicity (sulfamethazine, oxytetracycline, furazolidone), mutagenicity, nephropathy (gentamicin), hepatotoxicity, reproductive disorders, bone marrow toxicity (chloramphenicol), and even anaphylactic shock in humans. In addition, residues of antibiotics in milk are an issue of concern, since almost all classes of antibiotics that are used for humans are also used for food-producing animals [57,58].

The principle causes of the persistence of antibiotic residues in milk, according to Nikolić et al. [59], include non-compliance with the withholding period for antibiotic excretion from the animal body, overdose, use of banned antibiotics, and subsequent or deliberate addition of antibiotics to milk to prevent the multiplication of microorganisms that causes the deterioration of milk. Furthermore, for the treatment of mastitis, antibiotics are mostly administered through the intra-mammary route. Through this route, the active antibiotic reaches high concentrations at the infection site, being more effective at lower doses. However, the administered drug can be easily transferred from the mammary gland to the milk and is, therefore, another primary cause of the presence of residues in it. In addition, upon comparison to other cases where the antibiotics were administered parenterally, antibiotic residues in milk were found to persist for a longer period of time and in a higher concentrations, when the drug was given through the intra-mammary route [10]. In the current study, a certain degree of compliance was found among the Lebanese farmers to the international regulations in terms of the use of antibiotics and with respect to their withdrawal time.

After reviewing the literature, it was found that the data on the amount of antibiotics used in the dairy sector in Lebanon are scarce. There is little accurate information on antibiotic usage, the residues left in food products, and antibiotic resistance. Only a few studies could be found, and they only targeted one or two antibiotics in Lebanese milk. When compared to previous studies conducted in Lebanon, our results are consistent with Mokh et al. [60], in which tylosin was detected below MRLs; however, the concentration of oxytetracycline was found to exceed the MRL, unlike in the current study, where only one sample exceeded the MRL. In addition, Mokh et al. [60] reported the highest contamination of milk samples to be in Mount Lebanon, while in this study, it was found to be in Beqaa and Baalbeck–Hermel (Table 5 and Table 8). Those variations could be attributed to the difference in the tests used for the screening and their efficiency, the study time, and the sample size. Furthermore, our results are also similar to those of Kassaify et al. [61], who also reported a mean residue level of gentamicin below the MRL (56.25 μg/kg); however, this value was higher than that detected in our study.

### 4.1. Comparison with Other Countries

In comparison with other data concerning antibiotic residue in milk, the oxytetracycline concentration reported in our study was higher than that reported in Turkey (29.36 μg/kg) [62] and North Italy (5.82 μg/kg) [63]. On the other hand, it was less than that of Ardabil, Iran (40.0 μg/kg) [64]; Egypt (97.9 μg/kg in cow’s milk and 85.5 μg/kg in Buffalo’s milk) [65]; Bangladesh (61.29 μg/kg) [36]; Ethiopia (45–192 µg/kg) [66]; India (70.7 μg/kg) [67]; and the United States (34.21 μg/kg) [68].

With respect to gentamicin, a study in India [69] revealed that the mean residue level of gentamicin was 47.92 μg/kg, which is above the MRL and higher than that reported in our study (5.5 µg/kg). However, studies in Croatia (1.12 μg/kg) [70] and Iraq (in winter = 1.247 μg/kg; in spring = 3.225 μg/kg) [71] reported mean residue levels of gentamicin less than those obtained in our study.

As for colistin and tylosin, insufficient data were available concerning their occurrence in raw milk. In the present study, tylosin residues had a mean concentration of 4.44 μg/kg, which is higher than that reported in one study in Croatia (0.98 μg/kg in 2008, 0.62 μg/kg in 2009, and not detected in 2010) [70].

### 4.2. Risk Assessment to the Exposure of the Lebanese Population to Antibiotic Residues from Raw Milk

To the best of our knowledge, this is the first study in the literature to assess the EDI, HQ, and % ADI of antibiotic residues in milk for both genders across all age groups, which included early childhood, middle childhood and adolescents, adults, and elderly. In the current study, the deterministic approach resulted in a higher exposure estimation across all age groups for all antibiotics detected. This could be due to the fact that milk intake reported by FAOSTAT was higher than that obtained from the national studies. Those findings are in line with a study conducted in Iran, in which the deterministic approach resulted in an estimated daily exposure exceeding that obtained from the probabilistic approach. However, the antibiotic under investigation in that study was tetracycline [35].

Due to the scarcity of data in the literature regarding other age groups, the average EDI among adults for each antibiotic in the current study was compared to others obtained worldwide. Upon comparing the mean EDI of oxytetracycline obtained through the deterministic method with other studies, we found that it was higher than that reported in Bangladesh (0.168 μg/kg/day [36]. On the other hand, if the probabilistic estimation was considered for the same antibiotic, the value reported in our study would be lower. This was also applicable in another study, conducted in Croatia [70], which reported EDI values for gentamicin (0.073 μg/kg b.wt/day) and tylosin (0.044 μg/kg b.wt/day). On the other hand, both estimations obtained in the current study were below the EDI values for gentamicin in Gujarat (4.2 μg/kg b.wt/day) and India (0.24 μg/kg b.wt/day), which were presented in the same study [69].

The hazard quotient (HQ) of the detected antibiotics was below one among both genders in all age groups. This shows that the levels of antibiotic residues detected in the raw bovine milk samples in the present study had negligible toxicological effects on the health of the consumers. Those findings were similar to other studies in Bangladesh (oxytetracycline), Gujarat (gentamicin), and India (gentamicin), which were all below 1.

### 4.3. Microbiological Quality of Milk

This study revealed a significant contamination of milk samples with mesophilic aerobes with all 30 samples reporting a mean value greater than the permitted maximum value established by the Lebanese norm (LIBNOR). Mesophilic aerobes are usually considered to be an indicator microorganism used to identify the microbiological quality of milk and dairy products. Therefore, we can come to an understanding that there are poor sanitary and hygienic conditions of milking, storage, and processing of milk in Lebanon. This high level of contamination can also propose a probability that there might be pathogenic microorganisms present in the milk [72].

According to our statistics and according to the data published by the LACTIMED group, a project funded by the European Union [73], the kind of farms in our study do not frequently use electrical machines for milking cattle. Moreover, nowadays, in Lebanon, due to the financial crisis and the fuel shortage, most farmers and farm owners are struggling with the shortage in electricity, which limits the use of milking machines. In the present study, four milk samples were found contaminated with *Escherichia coli*, the gut bacteria. According to the literature, the main causes of such contamination include fecal contamination, poor hygienic status during manual milking, unsafe milking equipment/collection tanks, and intramammary contamination during clinical mastitis [24].

In raw milk, the presence of *E. coli* is a health issue due to its exposure to antibiotics, and thus it may develop resistance in order to survive. This is markedly demonstrated in our findings in which *E. coli* isolated from one of the samples indicated the occurrence of ESBL-resistant strain. ESBL is one of the antibiotic resistance mechanisms in which the bacteria releases specific enzymes (such as β-lactamase) to break down a specific atom ring of the antibiotics [74]. Similar findings were also reported in other studies conducted in Turkey [75] and Germany [76], which found the most frequent ESBL-producing species to be *E. coli*.

Another important finding in our study was the detection of three samples contaminated with *S. aureus* with mean concentrations exceeding that of the Lebanese norms [45]. In addition to being responsible along with *E. coli* for causing clinical and subclinical mastitis in cows, *S. aureus* pathogens can produce, under specific conditions, heat-stable enterotoxins, which are a serious cause of food poisoning [77]. One of the contaminated samples was found to be resistant to two antibiotics, penicillin and cefoxitin, thus representing a methicillin-resistant *S. aureus* strain. Like that of the ESBL, this is an alarming result since under specific conditions, these bacteria might become vectors of antibiotic resistance genes, transferring them to humans [78] and therefore leading to serious health implications among the Lebanese population. Moreover, the current findings *on S. aureus* come in line with others obtained in Iran [79], in which eight MRSA positive samples were identified, and North West India [80], where twenty-seven *S. aureus* isolates were found to be multidrug resistant, of which fifteen were methicillin resistant. Lastly, a study conducted in Northern Italy [81] found four multidrug-resistant isolates of *S. aureus*, which were classified as methicillin resistant. Infections caused by antibiotic-resistant organisms continue to add considerable and avoidable costs to the already overburdened Lebanese healthcare system. The infections lead to complications that require additional therapeutic interventions, including indwelling catheters, sophisticated life support, intravenous fluid therapy, and prosthetic devices. They can also extend the hospital stay and the use of broad-spectrum antibiotics appreciably, which in turn can increase the prevalence rate of multidrug-resistant pathogens. In Lebanon, the resistance trends of bacterial isolates of *E. coli* and *S. aureus* have been reported in many hospitals for several years. The prevalence rate of methicillin-resistant *S. aureus* was 27.6%, and the extended-spectrum beta-lactamase production rate of *E. coli* was 32.3% [82].

## 5. Strengths and Limitations

This is the first study in Lebanon to test for residues of 16 different antibiotics commonly used in livestock care. In addition, not only was the present study the first to perform a comprehensive risk assessment and risk characterization to antibiotic residues in milk among all age groups in Lebanon, but also, to our knowledge, this is the first study in the literature to perform exposure and risk assessment to antibiotic residues in raw bovine milk in both genders across all age groups. On the other hand, specific limitations accompanied the current study. First, the sample size was originally 400; however, due to the limited budget, only 200 milk samples were tested. In addition, the virulence profile of isolated bacteria was not investigated. Furthermore, due to the scarcity in the Lebanese data regarding the average weight for age, the information used to calculate the exposure among different age groups was based on the CDC charts. In addition, the gross exposure to antibiotic residues did not take into consideration additional food groups that might be contaminated as well with antibiotic residues. Consequently, studies that will be conducted in the future should focus more on collecting data concerning the Lebanese normal growth patterns and milk consumption. This way, more national data will be available and at hand to assess the exposure to antibiotic residues through milk and various dairy products, as these food components occupy an important place in the Lebanese diet. The assessment of the emergence and dissemination of antibiotic resistance in raw milk and other environments in Lebanon would benefit greatly from future studies on the underlying genetic mechanisms of resistance.

## 6. Conclusions

In conclusion, the present study validated that the raw milk produced in the different governorates in Lebanon does not violate the MRLs established by the FAO/WHO. However, the persistence of antibiotic residues in milk is reported to contribute to the development of antibiotic resistance, as was clearly shown in our study, which is an alarming result. It is especially important to control the contamination of milk through the employment of various prevention strategies. Some of these include an ongoing assessment and examination of the health of the cattle to avoid the spread of infection, increasing awareness among farmers about antibiotic residues and their potential health effects, and the provisions of guides for the farmers that demonstrate the proper usage and withdrawal period required for the excretion of antibiotics [83,84,85]. Lastly, to tackle ABR, it is crucial to support the concept of the “One Health” approach that encompasses human, animal, plant, and environmental health [86].

## Figures and Tables

**Table 1:** Collection date, number of samples collected, and farm characteristics across the different Lebanese governorates from which the samples were taken.

Governorate	Collection Date	Number (%) of Collected Samples	Number of Cattle per Farm per Governorate	Number of Subsistence Systems (1 to 3 Cows) ^a^	Number of Diverse Systems (4 to 6 Cows) ^b^	Number of Specialized Dairy Cattle Systems (7 or More Cows) ^c^
Beqaa and Baalbeck–Hermel	August 2018 March 2019 July 2019 August 2019 September 2019	121 (60.5%)	21	14	22	83
North Lebanon ^d^	April 2018 August 2019	22 (11%)	21	0	2	9
South Lebanon ^e^	July 2019	26 (13%)	15	0	1	21
Mount Lebanon	July 2019	31 (15.5%)	17	1	5	23
Total		200	74	15	30	136

^a^: Form of farming in which nearly all of the crops or livestock raised are used to maintain the farmer and the farmer’s family, leaving little, if any, surplus for sale or trade. ^b^: Share a common focus on local production, agro-ecological and local knowledge, and whole systems approaches to agriculture, based on promoting ecological diversity and ecosystem services from field to landscape scales. ^c^: The specialized dairy farms often use the agricultural area for the production of their own feeds and bedding. ^d^: North Lebanon includes Akkar. ^e^: South Lebanon includes Nabatiyeh.

**Table 2:** Liquid chromatography and mass spectrometry analytical performance for antibiotics.

Compound	CCα (µg.kg^−1^) Decision Limit	CCβ (µg.kg^−1^) Detection Capability	Recovery %	CV Intraday %	CV Interday %
Enrofloxacin	0.11	0.24	97	11	13
Marbofloxacin	0.14	0.25	96	10	12
Trimethoprim	0.25	0.33	95	10	14
Gentamicin	0.23	0.31	96	11	16
Sulfamethazin	0.11	0.23	95	10	12
Oxytetracycline	0.10	0.24	97	10	14
Florfenicol	0.22	0.32	95	15	18
Tilmicosin	0.13	0.25	95	8	12
Spectinomycin	0.35	0.51	95	13	18
Penicillin G	0.15	0.30	96	12	16
Amoxicillin	0.51	0.65	95	15	18
Spiramycin	0.12	0.25	95	11	13
Colistin	0.20	0.30	95	10	13
Kanamycin	0.50	0.70	95	9	12
Danofloxacin	0.13	0.27	95	11	13
Tylosin	0.15	0.25	96	12	15

**Table 3:** Distribution of cattle diseases across the Lebanese governorates.

Disease	Number of Infected Cattle	Governorate
Cowpox–septicemia	7	Mount Lebanon
FMD ^a^	16	Beqaa + Baalbeck–Hermel
Hepatitis	25	South
Hepatitis–mastitis	56	South
Hepatitis–mastitis–respiratory infection	41	South
Hoof disease	24	Mount Lebanon
Non-specific infections	14	Mount Lebanon
Mastitis–hoof disease	40	Beqaa + Baalbeck–Hermel

^a^ Foot and mouth disease.

**Table 4:** Residual levels of the 16 tested antibiotics in collected raw milk samples.

Antibiotic	Positive Samples N (%)	Minimum Value (μg/kg)	Maximum Value (μg/kg)	Mean * ± SD ^a^ (μg/kg)	MRL ^b^ Value (μg/kg)	No. of Samples Exceeding MRLs	References
Oxytetracycline	16 (8)	5.19	123.73	31.51 ± 13.23	100	1	[34]
Colistin	4 (2)	2.15	8.33	4.56 ± 0.73	50	0	[33]
Tylosin	8 (4)	3.07	5.75	4.44 ± 0.89	100	0	[33]
Gentamycin	2 (1)	5.33	5.67	5.5 ± 0.55	200	0	[33]
Enrofloxacin	0 (0)	ND	ND	ND	100 **	ND	[38]
Marbofloxacin	0 (0)	ND	ND	ND	75	ND	[39]
Trimethoprim	0 (0)	ND	ND	ND	50	ND	[40]
Sulfamethazin	0 (0)	ND	ND	ND	25	ND	[33]
Florfenicol	0 (0)	ND	ND	ND	-	ND	-
Tilmicosin	0 (0)	ND	ND	ND	40	ND	[41]
Spectinomycin	0 (0)	ND	ND	ND	200	ND	[33]
Penicillin G	0 (0)	ND	ND	ND	4	ND	[33]
Amoxicillin	0 (0)	ND	ND	ND	4	ND	[33]
Spiramycin	0 (0)	ND	ND	ND	200	ND	[33]
Kanamycin	0 (0)	ND	ND	ND	100	ND	[42]
Danofloxacin	0 (0)	ND	ND	ND	100	ND	[43]

* Mean of the contaminated samples. ** Sum of enrofloxacin and ciprofloxacin. ^a^: SD: standard deviation. ^b^: MRL: maximum residue limits.

**Table 5:** Distribution of positive samples for antibiotic residues among the different Lebanese governorates.

Governorate	Oxytetracycline N (%)	Gentamicin N (%)	Colistin N (%)	Tylosin N (%)	Total Number of Contaminated Samples N (%)	Total Number of Contaminated Samples Collected from Each Governorate N (%)
Beqaa and Baalbeck–Hermel	9 (30)	2 (6.7)	4 (13.3)	7 (23.3)	22/30 (73.3)	22/121 (18.2)
North Lebanon	2 (6.7)	0	0	0	2/30 (6.7)	2/22 (9.1)
South Lebanon	2 (6.7)	0	0	0	2/30 (6.7)	2/26 (7.7)
Mount Lebanon	3 (10)	0	0	1 (3.3)	4/30 (13.25)	4/31 (12.9)
Total	16/30 (53.3)	2/30 (6.7)	4/30 (13.3)	8/30 (26.7)	30/30 (100)	30/200

**Table 6:** Risk characterization of dietary exposure to antibiotic residues through consumption of milk among various age groups using the deterministic approach.

Age Group		Weight (kg)	g/day	kg/day	Oxytetracycline			Colistin			Tylosin			Gentamycin		
					EDI	HQ	% ADI	EDI	HQ	% ADI	EDI	HQ	% ADI	EDI	HQ	% ADI
**Early** **childhood** **(≤5)**	Males	13.41	110.95	1.11	2.61	0.09	8.69	0.38	0.05	5.39	0.37	0.01	1.22	0.46	0.02	2.28
	Females	12.75	110.95	1.11	2.74	0.09	9.14	0.40	0.06	5.67	0.39	0.01	1.29	0.48	0.02	2.39
**Middle childhood and** **adolescents (6–19.9 years)**	Males	52	110.95	1.11	0.67	0.02	2.24	0.10	0.01	1.39	0.09	0.003	0.32	0.12	0.01	0.59
	Females	48.4	110.95	1.11	0.72	0.02	2.41	0.10	0.01	1.49	0.10	0.003	0.34	0.13	0.01	0.63
**Adults** **(20–59.9)**	Males	60	110.95	1.11	0.58	0.02	1.94	0.08	0.01	1.20	0.08	0.003	0.27	0.10	0.01	0.51
	Females	60	110.95	1.11	0.58	0.02	1.94	0.08	0.01	1.20	0.08	0.003	0.27	0.10	0.01	0.51
**Elderly** **(≥60 years)**	Males	78.88	110.95	1.11	0.44	0.01	1.48	0.06	0.01	0.92	0.06	0.002	0.21	0.08	0.004	0.39
	Females	71.68	110.95	1.11	0.49	0.02	1.63	0.07	0.01	1.01	0.07	0.002	0.23	0.09	0.004	0.43

EDI: estimated daily intake (μg/kg BW/day); HQ: hazard quotient; %ADI: percentage acceptable daily intake. Weights for age 0–2 years were taken from CDC growth charts (https://www.cdc.gov/growthcharts/html_charts/wtageinf.htm (accessed on 17 August 2022) and https://www.cdc.gov/growthcharts/data/set3/all.pdf (accessed on 17 August 2022)). The 75th percentile was used for the calculations. Children under 5 included children from the age of 7.5 months till 4 years. The intake of milk in grams was calculated according to the following rule: weight (g) = volume (mL) × density (g/cm^3^). Density of milk was considered as 1.03 g/cm^3^ [44]. ADI of oxytetracycline: 0–30 μg/kg; ADI of colistin: 0–7 μg/kg; ADI of tylosin: 0–30 μg/kg; ADI of gentamycin: 0–20 μg/kg [33].

**Table 7:** Risk characterization of dietary exposure to antibiotic residues through consumption of milk among various age groups using the probabilistic approach.

Age Group		Weight (kg)	g/day	kg/day	Oxytetracycline			Colistin			Tylosin			Gentamycin		
					EDI	HQ	% ADI	EDI	HQ	% ADI	EDI	HQ	% ADI	EDI	HQ	% ADI
**Early** **Childhood** **(≤5)**	Males	13.41	110.95	1.11	1.62	0.05	5.40	0.23	0.33	3.35	0.228	7.6 × 10^−3^	0.76	0.28	0.01	1.41
	Females	12.75	110.95	1.11	1.70	0.06	5.68	0.25	0.035	3.52	0.240	8 × 10^−3^	0.80	0.30	0.02	1.49
**Middle childhood and** **adolescents (6–19.9 years)**	Males	52	110.95	1.11	0.037	1.23 × 10^−3^	0.123	0.005	7.14 × 10^−4^	0.08	0.005	1.67 × 10^−4^	0.02	0.006	3 × 10^−4^	0.03
	Females	48.4	110.95	1.11	0.040	1.33 × 10^−3^	0.132	0.006	8.57 × 10^−4^	0.08	0.006	2 × 10^−4^	0.02	0.007	3.5 × 10^−4^	0.03
**Adults** **(20–59.9)**	Males	60	110.95	1.11	0.014	4.67 × 10^−4^	0.047	0.002	2.86 × 10^−4^	0.03	0.002	6.67 × 10^−5^	0.01	0.002	1 × 10^−4^	0.01
	Females	60	110.95	1.11	0.019	6.32 × 10^−4^	0.062	0.003	4.29 × 10^−4^	0.04	0.003	1 × 10^−4^	0.01	0.003	1.5 × 10^−4^	0.02
**Elderly** **(≥60 years)**	Males	78.88	110.95	1.11	0.013	4.33 × 10^−4^	0.043	0.002	2.86 × 10^−4^	0.03	0.002	6.67 × 10^−5^	0.01	0.002	1 × 10^−4^	0.01
	Females	71.68	110.95	1.11	0.020	6.67 × 10^−4^	0.065	0.003	4.29 × 10^−4^	0.04	0.003	1 × 10^−4^	0.01	0.003	1.5 × 10^−4^	0.02

EDI: estimated daily intake (μg/kg BW/day); HQ: hazard quotient; %ADI: percentage acceptable daily intake. Weights for age 0–2 years were taken from CDC growth charts (https://www.cdc.gov/growthcharts/html_charts/wtageinf.htm and https://www.cdc.gov/growthcharts/data/set3/all.pdf) (accessed on 17th August 2022). The 75th percentile was used for the calculations. Children under 5 included children from the age of 7.5 months till 4 years. The intake of milk in grams was calculated according to the following rule: weight (g) = volume (mL) × density (g/cm^3^). Density of milk was considered as 1.03 g/cm^3^ [44]. ADI of oxytetracycline: 0–30 μg/kg; ADI of colistin: 0–7 μg/kg; ADI of tylosin: 0–30 μg/kg; ADI of gentamycin: 0–20 μg/kg [33].

**Table 8:** Prevalence of microbial contamination by mesophilic flora, *E. coli*, and *S. aureus* of raw milk samples with antibiotic residues according to the farm locations, and the number and the health issues of cattle per farm.

Samples	No. of Cattle/Farm	Date of Collection	Disease	Governorates ^a^	Oxytetracycline Mean (μg/kg)	Colistin Mean (μg/kg)	Tylosin Mean (μg/kg)	Gentamicin Mean (μg/kg)	Mesophilic Flora at 30 °C (CFU/mL)	*E. coli* at 42 °C (CFU/mL)	*S. aureus* at 37 °C (CFU/mL)
B126	45	Jul-19	No	B-H	ND	ND	3.4	ND	0.80 × 10^5^	ND	ND
B27	10	Jul-19	No	B-H	ND	ND	ND	5.67	1.56 × 10^5^	2 × 10^2^	ND
B120	7	Jul-19	No	B-H	ND	8.33	ND	ND	3.60 × 10^5^	ND	ND
B11	7	Jul-19	No	B-H	ND	5.47	ND	ND	1.20 × 10^5^	ND	ND
B19	30	Jul-19	No	B-H	7.35	ND	ND	ND	1.13 × 10^5^	ND	ND
B72	4	Jul-19	No	B-H	ND	ND	5	ND	1.76 × 10^5^	ND	ND
B56	5	Jul-19	No	B-H	50.17	ND	ND	ND	0.76 × 10^5^	ND	ND
B51	30	Jul-19	No	B-H	11.06	ND	ND	ND	0.48 × 10^5^	3.4 × 10^2^	ND
B37	16	Jul-19	No	B-H	ND	ND	4.08	ND	2.08 × 10^5^	ND	ND
B314	5	Sep-19	No	B-H	14.36	ND	ND	ND	1.20 × 10^5^	ND	ND
B143	70	Jul-19	No	B-H	75.98	ND	ND	ND	2.16 × 10^5^	3 × 10^2^	5 × 10^2^
B159	14	Aug-19	No	B-H	ND	ND	ND	5.33	2.36 × 10^5^	ND	ND
B171	40	Aug-19	No	B-H	10.39	ND	ND	ND	2.80 × 10^5^	ND	ND
B253	8	Jul-19	No	B-H	ND	ND	5.03	ND	3 × 10^4^	ND	ND
B252	22	Sep-19	No	B-H	ND	2.28	ND	ND	2.80 × 10^5^	ND	ND
B200	17	Aug-19	No	B-H	61.21	ND	ND	ND	3.20 × 10^5^	ND	ND
B254	25	Sep-19	No	B-H	16.1	ND	5.75	ND	2.24 × 10^5^	ND	ND
B240	40	Aug-19	No	B-H	ND	ND	3.95	ND	2.12 × 10^5^	ND	ND
B258	19	Sep-19	No	B-H	ND	ND	3.07	ND	2 × 10^4^	ND	ND
B234	25	Aug-19	No	B-H	24.63	ND	ND	ND	0.80 × 10^5^	ND	160
B188	8	Aug-19	No	B-H	6.65	ND	ND	ND	1.68 × 10^5^	ND	ND
ML12	25	Jul-19	No	ML	ND	ND	5.24	ND	2.20 × 10^5^	ND	ND
ML55	8	Jul-19	No	ML	8.52	ND	ND	ND	0.88 × 10^5^	ND	ND
ML37	6	Jul-19	No	ML	21.27	ND	ND	ND	2.08 × 10^5^	ND	ND
ML28	10	Jul-19	No	ML	9.82	ND	ND	ND	2.40 × 10^5^	ND	8.4 × 10^2^
N114	18	Aug-19	No	N	72.32	ND	ND	ND	3.20 × 10^5^	ND	ND
N108	10	Aug-19	No	N	42.18	ND	ND	ND	2.40 × 10^5^	ND	ND
S79	18	Jul-19	No	S	6.28	ND	ND	ND	2.20 × 10^5^	ND	ND
B83	35	Jul-19	No	B-H	123.73	ND	ND	ND	1.40 × 10^5^	4 × 10^2^	ND
B156	20	Jul-19	No	B-H	ND	2.15	ND	ND	2.40 × 10^5^	ND	ND

ND: not detected; maximum tolerable value according to European Norms: mesophilic flora = 104–105, *E. coli* = 102–103, *S. aureus* = 101–102 [46]. **^a^**: Kruskal–Wallis test was used to investigate the difference in antibiotics use between governorates; *p* = 0.007.
